# Effects of long-term continuous cropping on soil nematode community and soil condition associated with replant problem in strawberry habitat

**DOI:** 10.1038/srep30466

**Published:** 2016-08-10

**Authors:** Xingyue Li, Edwin E. Lewis, Qizhi Liu, Heqin Li, Chunqi Bai, Yuzhu Wang

**Affiliations:** 1 Department of Entomology, China Agricultural University, Beijing 100193, China; 2 Institute of Plant Protection, Sichuan Academy of Agricultural Science, Chengdu 610066, China; 3 Department of Entomology & Nematology, University of California-Davis, Davis, CA 95616, Untied States; 4 Institute of Pomology and Forestry, Beijing Academy of Agriculture and Forestry Sciences, Beijing 100093, China

## Abstract

Continuous cropping changes soil physiochemical parameters, enzymes and microorganism communities, causing “replant problem” in strawberry cultivation. We hypothesized that soil nematode community would reflect the changes in soil conditions caused by long-term continuous cropping, in ways that are consistent and predictable. To test this hypothesis, we studied the soil nematode communities and several soil parameters, including the concentration of soil phenolic acids, organic matter and nitrogen levels, in strawberry greenhouse under continuous-cropping for five different durations. Soil pH significantly decreased, and four phenolic acids, i.e., p-hydroxybenzoic acid, ferulic acid, cinnamic acid and p-coumaric acid, accumulated with time under continuous cropping. The four phenolic acids were highly toxic to *Acrobeloides* spp., the eudominant genus in non-continuous cropping, causing it to reduce to a resident genus after seven-years of continuous cropping. Decreased nematode diversity indicated loss of ecosystem stability and sustainability because of continuous-cropping practice. Moreover, the dominant decomposition pathway was altered from bacterial to fungal under continuous cropping. Our results suggest that along with the continuous-cropping time in strawberry habitat, the soil food web is disturbed, and the available plant nutrition as well as the general health of the soil deteriorates; these changes can be indicated by soil nematode community.

Continuous-cropping is a common practice in intensive agricultural production, particularly for horticultural crops. However, continuous-cropping can negatively affect soil fertility and physicochemical properties, leading a decline in crop productivity^1^. Many crops, both perennial and annual, experience suppressed plant growth and decreased yield when repeatedly planted in the same site^2^. This well-known phenomenon is called replant problem, the mechanisms of which are complex, and have been associated with multiple cultural practices, soil conditions and other environmental factors^3^,^4^,^5^. These conditions are also often associated with accumulated populations of fungal pathogens and nematodes^6^.

Strawberry (*Fragaria ananassa* Duch.) is one of the typical annual plants and terribly threatened by replant problem when new seedlings are established on sites under continuous-cropping condition. Replant problem manifests in stunted growth, declined crop vigor, weak root systems, and drying foliage, all of which lead to low productivity and shortened economic life^6^,^7^. For continuously cropped strawberry, replant problem has been attributed to biotic and abiotic factors, including accumulated phytotoxic allelochemical substrates^8^,^9^,^10^, build-up of specific pathogenic microorganisms (fungi, bacteria and actinomycetes)^11^,^12^,^13^,^14^ and plant-parasitic nematodes^15^,^16^,^17^, as well as unbalanced availability of plant nutrients and other declines in soil health (soil acidification, etc.).

Autotoxicity, a special form of allelopathy which is caused by phytotoxic allelochemical substances released by preceding plants of the same species, plays a significant role in replant problem^18^,^19^. Phenolic acids, such as p-hydroxybenzoic and cinnamic acids, which exhibit the strongest inhibition of plant growth, have been detected in strawberry root exudates grown either hydroponically or in solid medium^8^,^10^,^20^. When phenolic acids accumulate in soil, not only can the rhizosphere micro-ecosystem could undergo complex changes, but soil enzyme activity and nutrient cycling may be affected as well^21^,^22^,^23^,^24^.

Nematodes are of particular interest because they are ubiquitous in the soil environment and occupy key positions in soil food webs^25^. As the most abundant groups of soil fauna, the soil nematode community includes genera at most trophic levels, and plays critical roles in controlling organic matter decomposition and nutrient cycling; thus affecting the availability of plant nutrients^26^,^27^. Therefore, nematodes are ideal bioindicators for terrestrial ecosystems and their community structure can provide important insights regarding many aspects of ecosystem function^28^. Since soil nematodes are food- and host-specific, and sensitive to anthropogenic disturbance, such as agricultural management activities^29^,^30^,^31^, their populations and community structure can be directly and indirectly effected by soil perturbations. Furthermore, changes in the soil environment that affect the stability of soil ecosystems can be measured using an array of ecological indices calculated from nematode community structure, including diversity indices and functional indices^32^,^33^,^34^. Specifically, Genus dominance (*Ig*) as well as Shannone-Weaver index (*H*′) may be used interchangeably to give the distribution of species abundance, and Trophic diversity index (*TD*) describes the diversity of functional groups within the nematode populations^35^,^36^,^37^, despite the value scale differences. At the end of the last century, nematode families were classified along colonizer-persister (cp) continuum, and then nematode faunal analyses were modified including the development of an enrichment structure weighting system and nematode channel ratio (NCR)^38^. The structure index and enrichment index were derived from weighted faunal analysis. *SI* is a measure of the number of trophic layers and potential for regulation of opportunists, whereas *EI* assesses food-web responses to available resources^33^. Nematode faunal analysis provides a powerful tool for diagnosis of the complexity and status of soil food webs^39^.

Although faunal analysis of nematode community structure can provide assessments of the structure, function, and probably the resilience of the soil ecosystem^29^,^39^, they have not been employed to monitor the changes that take place in soil that undergoes continuously cropped strawberry. Here we suggest that using nematode community structure as biological indicator of soil health can improve our understanding of the causal agents of replant problem from the viewpoint of the soil ecosystem. This study was intended to explore parallels and differences in biodiversity and structure of the soil nematode community after different durations of continuous-cropping of strawberry.

Therefore, we studied the variation and common patterns in soil nematode communities and several parameters of soil, including the concentration of soil phenolic acids, organic matter and nitrogen levels, in five strawberry greenhouse sites under continuous-cropping for different durations. As one of the more integrated studies on replant soil so far, this work addresses the following questions:(i)

- Wh at are the dominant nematode taxa in soil under continuous cropping of strawberry and do the dominant taxa change with time?
- How do continuous-cropping affect edaphic parameters, such as nutrient levels, soil pH, and phenolic acids in the strawberry habitat? Among different continuous-cropping durations, what are the main parameters structuring the patterns of soil nematode community?
- How do nematode community structure and biodiversity change with time in continuously cropped strawberry? Can the nematode community patterns and levels of diversity be used to predict and reflect the other soil changes?

## Materials and Methods

### Site descriptions

The experiment was conducted in strawberry farms in Haidian District, Beijing, China (38°54′N, 116°23′E). The average annual temperature is about 12.5 °C, and the average precipitation is 628.9 mm during the growing season. Strawberries were planted in greenhouses and all the greenhouses were located in the same field with identical soil type (loam) according to China’s soil classification retrieval system, and maintained under the same management practices. Five durations of continuous cropping were compared: non-continuous cropping (0-Y), two-years (2-Y), three-years (3-Y), five-years (5-Y) and seven-years (7-Y).

The experimental greenhouses were established following 18-years of peach cultivation. It was the first time to plant strawberry in the 0-Y greenhouses, while the greenhouses of other treatments had been used to plant strawberry for years respectively. Each greenhouse area was approximately 0.5–0.6 hm^2^, holding 60–80 strawberry beds, each of which was 100 cm length ×40 cm width ×40 cm height. Strawberry seedlings were planted in two rows per bed with 15 cm between plants. Soil sampling was conducted in three strawberry farms as three replicates. One greenhouse was selected randomly in each farm for each treatment (continuous cropping-duration), that means five greenhouses were conducted per experimental farm.

### Soil sampling

To monitor nematode populations, soil samples were taken four times during the strawberry growing seasons: Aug. 28, 2011 (Seedling period), Nov. 10, 2012 (Blooming period), Jan. 5, 2012 (Young fruiting period), and Feb. 12, 2012 (Fruit harvest period).

Three sub-plots were arranged in each greenhouse, and each sub-plot contained 9 beds with approximately 270 strawberry plants. Ten cores (5 cm diameter × 20 cm deep) were collected from each subplot with a soil auger (diameter, 25 mm) in a Z-pattern in each sub-plot, approximately 5 cm from the nearest strawberry plant to a depth of 20 cm. Thus, a total of 135 samples (3 replicates × 5 treatments × 3 greenhouses × 3 sub-plots) were collected on each sampling date. Samples from the same greenhouse (three sub-plots) were pooled for each sampling date, mixed and sieved (2 mm) and stored in individual plastic bags. Samples were immediately transferred to a cold room maintained at 4 °C and processed within one week.

### Soil environmental condition

The soil pH value was measured by glass electrode^40^. Soil organic matter content was determined by burning dried soil in a muffle furnace at 490 °C for 8 h. Total nitrogen was measured colorimetrically following micro-Kjeldahl digestion^41^. Soil microbial biomass nitrogen (MBN) was extracted using the fumigation-extraction method and the corresponding soil extracts were measured using a TOC analyser (Multi C/N 3000, Analytik Jena, Germany), and were calculated as the differences in filtrates between the fumigated and unfumigated soil^42^,^43^.

### Extraction and identification of nematodes

Nematodes were extracted from 100 g of fresh soil of each sample, using a washing-sieving sugar flotation and centrifugation procedure, described by Courtney *et al*.^44^. Extracted nematodes were killed at 60 °C and fixed in 5% Formalin acetic acid^45^.

For each soil sample, 300–500 nematode individuals were identified to genus level using diagnostic keys^46^,^47^,^48^ under a Leica compound microscope (400×). Each nematode was assigned to one of the four trophic groups: bacterivores, fungivores, omnivores/predators, and herbivores^49^.

### Ecological indices

Nematode density was recorded as the total number of individuals per 100 g dry soil. We also calculated the following diversity indices: Genus dominance (*Ig*)^35^, Trophic diversity index (*TD*)^50^, Shannon-Weaver index (*H′*)^36^; and the following functional indices: Nematode channel ratio (*NCR*). Basal index (*BI*), Channel index (*CI*), Enrichment index (*EI*) and Structure index (*SI*)^33^ which were used to assess soil food web status^26^. Nematode genera were also assigned “c–p” values of 1–5, corresponding to their positions along the colonizer-persister continuum of their life histories^29^,^51^.

### Determination of Phenolic Acid Composition

Phenolic acids were extracted from soil by shaking with alkali^52^, and then measured by high-performance liquid chromatography (HPLC) at 245 nm, using Agilent 1100 LC/MSD Trap VL system (Agilent, USA) with a Symmetry® C_18_ HPLC column. (2.1 mm × 10 cm × 3.5 μm). Additional confirmations of the identities of the extracted phenolic acids were obtained with paper chromatography and by UV spectral comparisons in ethanol and ethanol plus sodium hydroxide^19^,^53^.

### Nematicidal toxicity bioassay

Nematodes used in these bioassays (*Acrobeloides* spp. and *Rhabditis* spp.) were isolated from the soil samples, and cultured on NGM (nematode growth medium) plates according to Ferris^54^. NGM plates were made as following steps: 3 g NaC1, 2.5 g Bactopeptone (Difco) and 17 g Bacto-agar (Difco) were dissolved in 975 ml distilled water; after autoclaving, 1 ml cholesterol in ethanol (5 mg/ml), 1 ml M CaCl_2_, 1 ml M MgSO_4_ and 25 ml M potassium phosphate buffer (pH 6.0) are added in order^55^.

Range-finding studies were run to determine the appropriate testing concentrations. A serial dilution of each of the four phenolic acids (five concentrations each) was prepared in distilled H_2_O. Nematode suspensions (20 μl) containing 100 juveniles were transferred to vials to which 980 μl of the phenolic acid solution was added. The vials were stored at 25 °C. Dead nematodes were counted daily for 72 h. After the last count, the inactive juveniles were maintained in distilled H_2_O for 24 h to test for their revival. Six repetitions for each treatment were performed using distilled H_2_O as control. Each entire experiment was conducted three times.

### Data analysis

The values of five treatments compared in this paper are the grand means from the samples of different periods during the strawberry growing season (3 replicates × 4 sampling times, 12 samples) with standard deviations. Data were analyzed by one-way ANOVA followed by Duncan’s multiple range tests using the SPSS statistical software Version 17.0 (SPSS Inc., Chicago, IL, USA). Differences at the P < 0.05 level were considered to be statistically significant. Results of nematicidal toxicity bioassay for the four phenolic acids were subjected to probit analysis using the PriProbit Program V1.6.3 to determine LC_50_ (median lethal concentration) values and their 95% confidence intervals (CI 95%)^56^.

Redundancy analysis (RDA) was performed using CANOCO 4.5 software (Ithaca, NY, USA) to determine the relationships between soil nematode communities and environmental parameters, and the Monte Carlo permutation test (n = 499) was employed to determine the significance of first canonical axis and all canonical axes^57^. Linear regression was performed in SPSS 17.0 software to determine relationships between soil nematode communities and environmental parameters.

## Results

### Soil environmental condition

**Figure 1:**
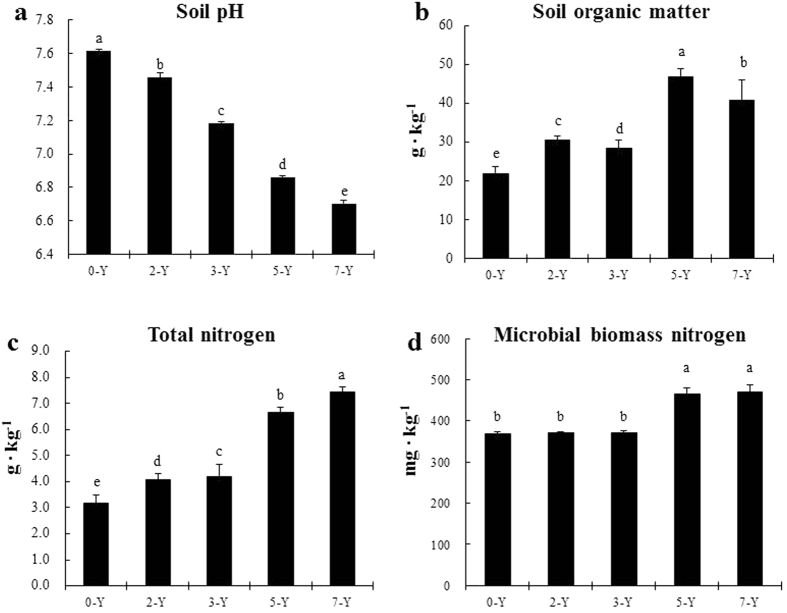
The effects of different years’ continuous-cropping on strawberry soil properties such as soil pH (**a**), soil organic carbon concentration (g C kg^−1^ soil) (**b**), total soil nitrogen concentration (g N kg^−1^ soil) (**c**) and microbial biomass nitrogen (mg ∙ kg^−1^) (**d**). Bars indicate standard deviation. Different letters indicate significant differences among different years of continuous cropping strawberry (ANOVA: Duncan test; *P* < *0.05*).

Soil pH gradually decreased with the continuous planting strawberry, and the pH value in 7-Y was 10% lower than that in non-continuous plantings (Fig. 1a). The concentration of organic matter generally increased through the continuous cropping years (Fig. 1b). The total nitrogen concentration increased with continuous cropping years (Fig. 1c); nevertheless, the microbial biomass nitrogen did not show a similar increase. Indeed after 5 years, N levels did not change (Fig. 1d).

### Phenolic acid composition

**Figure 2:**
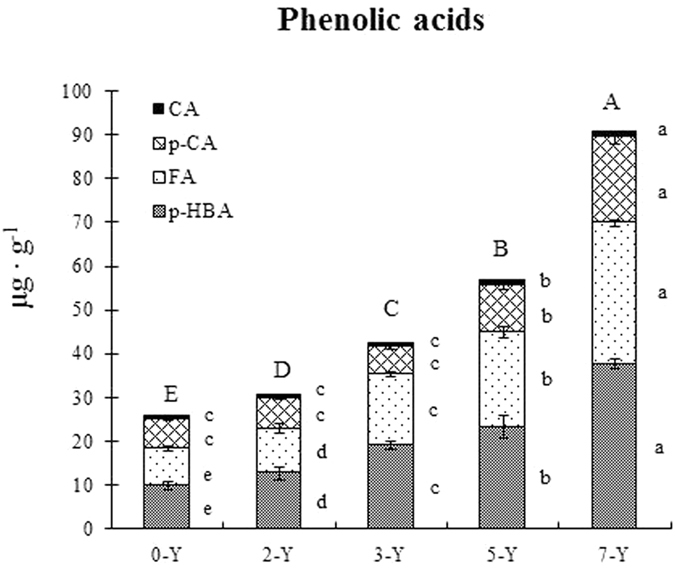
The effects of different years’ continuous-cropping on strawberry soil phenolic acids concentration (μg ∙ g^−1^). p-HBA, p-hydroxybenzoic acid; FA, ferulic acid; p-CA, cinnamic acid; CA, p-coumaric acid. Means followed by the same lowercase letters within the same phenolic acid/the same uppercase letters within total phenolic acid are not significantly different (ANOVA: Duncan test; *P* < *0.05*).

Four phenolic acids, i.e., p-hydroxybenzoic acid, ferulic acid, cinnamic acid and p-coumaric acid were detected in all soil samples with different years of continuous-cropping strawberry (Fig. 2). The content of p-hydroxybenzoic acid was the highest, cinnamic acid was the lowest (*P* < *0.05*). As the years of continuous-cropping increased, the concentration of the four phenolic acids increased significantly (*P* < *0.05*) in strawberry soil, especially for p-hydroxybenzoic acid (Fig. 2).

### Nematode composition

Twenty-eight genera of nematodes in eighteen families were found in total in all plots; five eudominant genera were identified (Table 1). Except for *Diploscapter* in the 7-Y ecosystem, *Rhabditis* and *Diploscapter*, which belong to the Ba_1_ guild, were eudominant genera in all greenhouses. *Eucephalobus* and *Acrobeloides* (Ba_2_) were eudominant genera only in 0-Y and 2-Y greenhouses. *Aphelenchoides* (Fu_2_) and *Tylenchus* (PF_2_) were only eudominant in 7-Y greenhouses (Table 1). The total number of nematodes extracted from the 0-Y greenhouses was significantly higher than other greenhouses (Table 1). Bacteriverous nematodes were the most dominant trophic group in each treatment, and comprised more than 55% of the total nematode population. Generally, the absolute abundance of herbivore nematodes first decreased slightly then increased substantially during the last three years (*P* < *0.05*). The population of fungivorous nematodes stayed stable during the first three years but increased during the next two years. The number of omniovores/predators doubled in the first two years, but declined quickly in the following years (Table 1).

### Nematode diversity indices

**Figure 3:**
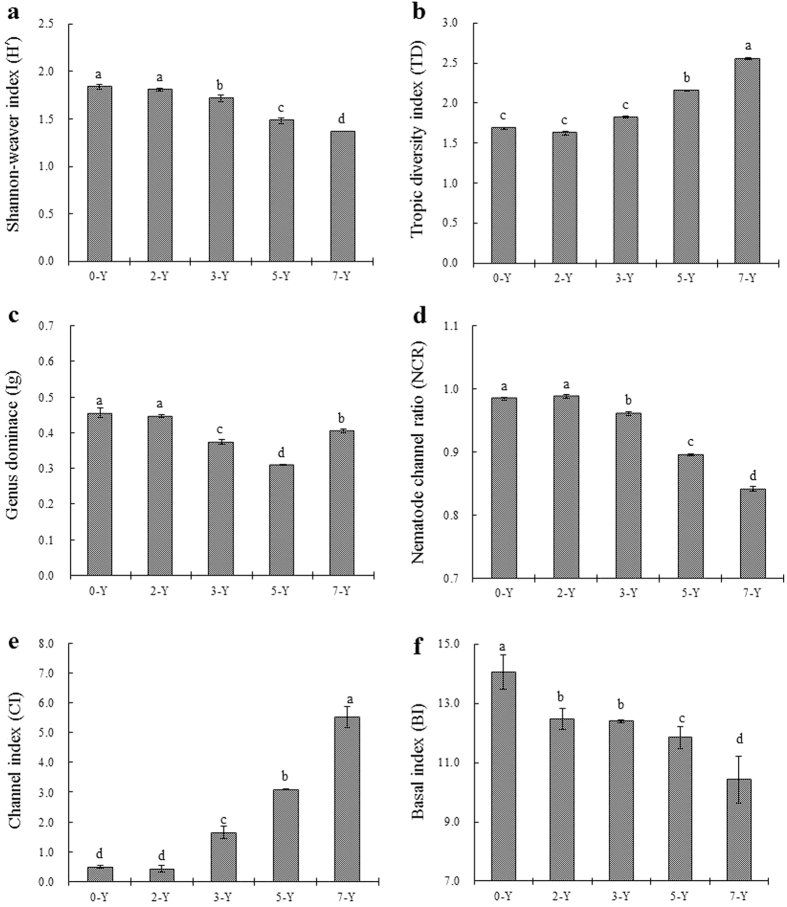
The effects of different years’ continuous-cropping on diversity and function of soil nematode communities in strawberry greenhouses. (**a**) Shannon-weaver diversity, (**b**) Trophic diversity, (**c**) Genus dominance, (**d**) Nematode channel ratio, (**e**) Channel index, (**f**) Basal index. Means within the same index followed by the same letters are not significantly different (ANOVA: Duncan test; *P* < *0.05*).

The *H′, TD*, and *Ig* indices in differed among years of continuous-cropping strawberry as shown in Fig. 3 (P < 0.05). The mean values of *H′* (Fig. 3a) and *Ig* (Fig. 3c) in both 0-Y and 2-Y sites were similar, and both were higher than other three sites. However, *TD* values remained low and consistent during the first three years, and then increased during the last two years (Fig. 3b).

### Nematode functional indices

The nematode channel ratio (*NCR*) in the first three years had high values, greater than 0.90, then they decreased from 5-Y to 7-Y (Fig. 3d). CI values were very low during the first two years, below 1.0, then climbed quickly and became significantly higher (above 6.0) in 7-Y (*P* < *0.05*) (Fig. 3e).

**Figure 4:**
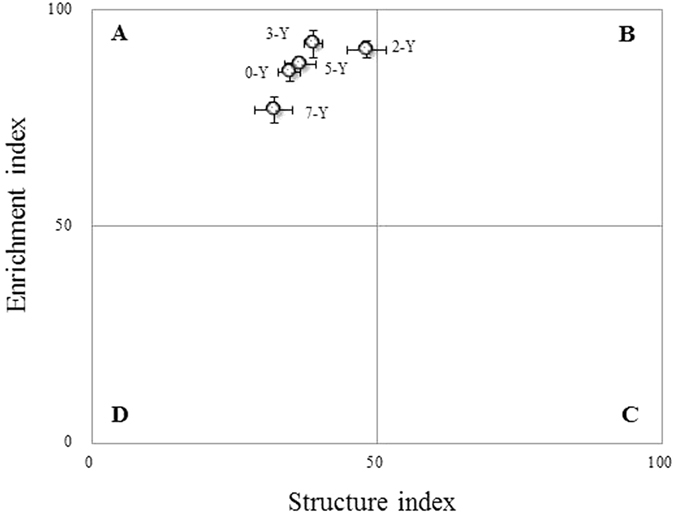
Faunal profiles representing the structure and enrichment conditions of different continuous-cropping years’ strawberry greenhouses.

The soil food webs among the strawberry sites were plotted along their respective *SI* and *EI* trajectories in Fig. 4. The nematode assemblages along the gradient all mapped to quadrant A, and no discernible pattern was observed in the nematode faunal profile.

### Nematicidal toxicity of phenolic acids

The four phenolic acids were toxic to both *Rhabditis* and *Acrobeloides* (Table 2). Cinnamic acid was the most potent nematicide against the two genera of nematodes. Moreover, all of the four phenolic acids possessed stronger toxicity against *Acrobeloides* (lower LC_50_ values) than *Rhabditis*, especially for ferulic acid and cinnamic acid, which had half the LC_50_ against *Acrobeloides* compared with *Rhabditis* (Table 2).

### Relationship between soil nematode communities and environmental parameters

**Figure 5:**
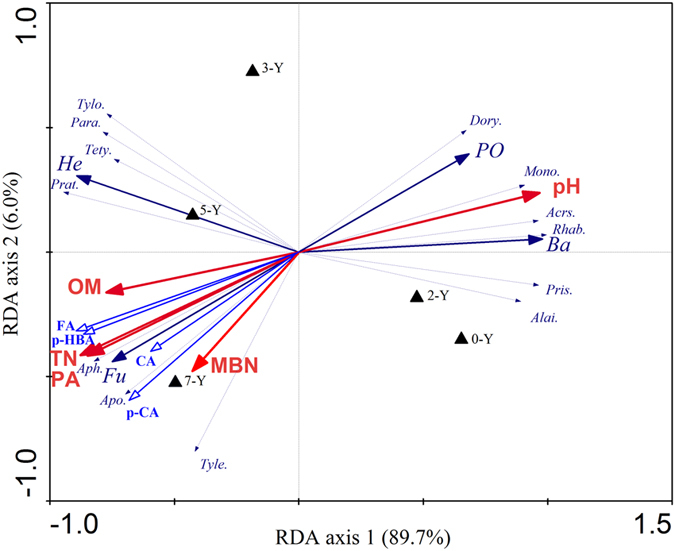
Redundancy analysis (RDA) diagram of the relation between soil nematode communities and environmental parameters (F = 63.324, P = 0.001). ▲Strawberry continuous-cropping duration; → Environmental parameters; ---→ Nematode genus or group; pH, Soil pH value; OM, Organic matter; TN, Total nitrogen; MBN, Microbial biomass nitrogen; Ba, Bacterivore; Fu, Fungivore; Om, Omniovores/predators; PF, Plant feeders; *Rhab., Rhabditis*; *Acrs., Acrobeloides*; Apo., *Aphelenchoides*; Aph., *Aphelenchus*; Pris., Prismatolainus; Alai., *Alaimus*; Dory., *Dorylaimus*; Mono., *Mononchus*; Para., *Paratylenchus*; Tety., *Tetylenchus*; Tyle., *Tylenchus*; Prat., *Pratylenchus*; Tylo., *Tylenchorhynchus.*

Direct ordination of the sites (RDA) had eigenvalues of 0.897 for the first axis and 0.06 for the second axis (Fig. 5). The Monte Carlo permutation test determined the first canonical axes (F = 43.240, *P* < *0.05*), and all canonical axes together (F = 63.324, *P* < *0.05*), explained a significant part of the variance in the dataset. RDA analysis results showed that different environmental parameters were positively or negatively correlated with the nematode communities in different strawberry continuous-cropping duration. Generally, the phenolic acids concentration correlated negatively with bacterivores (R^2^ = 0.749, *P* < *0.05*), predators and omnivores (R^2^ = 0.639, *P* < *0.05*), but correlated positively with fungivores (R^2^ = 0.940, *P* < *0.05*) (Fig. 5). Particularly, *Acrobeloides* (R^2^ = 0.812, *P* < *0.05*) and *Rhabditis* (R^2^ = 0.691, *P* < *0.05*) correlated negatively with phenolic acids concentration. Omniovores/predators such as *Dorylaimus* (R^2^ = 0.891, *P* < *0.05*) and *Mononchus* (R^2^ = 0.933, *P* < *0.05*) were correlated negatively with rich nutrient resources, at the same time, fungivores such as *Aphelenchoides* (R^2^ = 0.842, *P* < *0.05*) and *Aphelenchus* (R^2^ = 0.973, *P* < *0.05*) correlated positively with rich nutrient resources.

## Discussion

### Soil nematode composition and dominance

Although soil nematodes dominance differed among years of continuous-cropping strawberry, the composition of nematodes genera was similar because of the local pedogenesis and species pool, as well as the same plant habitat and same agronomic management. The Ba_1_ guild, *Rhabditis* and *Diploscapter*, were most dominant genera in all strawberry greenhouses. While *Eucephalobus* and *Acrobeloides* (Ba_2_) were eudominant genera only in 0-Y and 2-Y greenhouses, *Aphelenchoides* (Fu_2_) and *Tylenchus* (PF_2_) were only eudominant in 7-Y greenhouses.

Rhabditid nematode populations seem to be affected predominantly by sudden flushes of food resources^58^,^59^,^60^,^61^. Typically, organic matter addition triggers a quick increase of bacterial populations followed immediately by an increase of bacterivorous nematodes, mainly Rhabditidae as strict colonizers (Ba_1_)^32^. But bacteria populations, and then Ba_1_ nematode populations usually decline to previous or even lower population levels. This is followed by an increase of Ba_2_ nematode populations that feed more deliberately and continue feeding as resources decline. Generally *Rhabditis* and *Acrobeloides* were co-existing genera, which had a significant effect on ecosystem function through the creation of additional consumer, decomposer, and symbiotic organism niches^62^. *Aphelenchoides* were the most common fungivorous nematodes, and increased more than *Aphelenchus* through the years in the strawberry habitat. This can be explained by a previous study which indicated that *Aphelenchoides* is typically a more common fungivore, and may have less specialized feeding habits than *Aphelenchus*^37^. However, extremely higher high-density populations of *Aphelenchoides* dose harm to strawberry plants, because some strawberry nematodes belong to *Aphelenchoides* genus, such as *A. fragariae* which could terribly damage the strawberry leaves^63^. On the other hand, the high-density populations of herbivore nematodes, especially the He_2_ group, in 3-Y, 5-Y and 7-Y may result from accumulation year after year combined with the old root residues which were not completely removed. This is consistent with the fact that older systems had larger relative densities of root-parasitic nematodes^64^ and plant feeding nematodes could increase with time during succession in pathogen loads^65^. We speculate that the steady increase in plant feeding nematodes might be an important negative result of the continuous cropping of strawberry in greenhouse conditions.

### Edaphic parameters in related to soil nematode community

The amount of time that greenhouse strawberries are in continuous production impacted all soil environmental conditions in this study. Obviously, prolonged and repeated fertilizer application could lead to accumulation of organic matter, increased total nitrogen, and significantly decreased soil pH^30^. However we did not record an increase in microbial biomass nitrogen as significant as that in the total nitrogen, which suggested that the N supplying ability of soil as well as N uptake by plants didn’t increase remarkably^66^. Consequently, the increase of bacterial populations was short-lived as soon as easily decomposable substrates diminish, and populations of bacterivorous nematode populations declined. Worst of all, the nitrogen inputs exceeded that which could be used by soil microorganisms and the plants, and the extra nitrogen remained in the soil and caused soil acidification. The results are in line with other studies which also showed that soil pH was significantly decreased due to accumulated nitrogen^67^,^68^,^69^. Soil pH also may drop partly because strawberry, like many other crops, secretes phenolic acids through their roots, which accumulate in continuous-cropping conditions^70^. Here, in all soil samples from the 5 different continuous-cropping durations, four phenolic acids, i.e., p-hydroxybenzoic acid, ferulic acid, cinnamic acid and p-coumaric acid accumulated steadily. Apart from the acidification effect on soil, phenolic acids, which are phytotoxic, play an important role in allelopathic effects between strawberry plants in the replant ecosystem^71^,^72^,^73^. The accumulation of phenolic acids in soil could affect the rhizosphere micro-ecosystem, soil enzyme activity and nutrient cycling^21^,^22^,^23^,^24^. In addition to phenolic acids themselves, low soil pH may inhibit ions, such as NO_3_^−^, H_2_PO^4−^, and SO_4_^2−^, being absorbed by the plant root^74^, and the inhibition of ion uptake is partly responsible for the growth inhibition by allelochemicals.

Interactions at the aboveground-belowground interface provide important feedbacks that regulate ecosystem processes, and the resulting changes in soil properties might cause direct or indirect influences on below-ground soil fauna via ecological process, such as nutrients recycling and plant growth^75^. In this study, *Acrobeloides* (Ba_2_), decreased from a eudominant genus in 0-Y to a subdominant genus in 5-Y and further decreased to resident level in 7-Y. Whereas, *Rhabditis* (Ba_1_) remained the eudominant genus throughout all years. There are two potential explanations for this paradox. First, concentrations of the four phenolic acids in soil are approximately twice as high in 5-Y and more than three times higher in 7-Y, compared with 0-Y; the phenolic acids were more toxic to *Acrobeloides* than *Rhabditis*. Second, the continuous input of organic matter and nitrogen supplied *Rhabditis* (Ba_1_) with constant food resources that did not diminish as they would in a natural ecosystem. Furthermore, the RDA analysis result showed that soil nematode communities of five different durations under strawberry continuous-cropping significantly correlated with all the examined environmental factors, especially for nutrient resources together with phenolic acids which played the central role in structuring the patterns of the soil nematode community.

### Soil nematode biodiversity and function

Not just nematode community structure and biodiversity changed with time in continuously cropped strawberry, the community patterns and levels of diversity in soil for nematode reflected the other soil changes. Organisms within soil food webs are involved in processes of decomposition and nutrient mineralization, and their abundance and activity have been linked to plant ecophysiological traits such as the quality and quantity of plant tissue^75^.

The use of ecological indices as indicators of ecosystem quality (e.g. diversity, stability, and resilience of nematode populations) has received increased attention over the last decade. Specifically, *Ig* weights common taxa, *H′* is more sensitive to rare taxa which makes it more effective in assessing nematode diversity in continuous cropping systems^34^,^35^,^36^. In our study, several ecological indices illustrated soil nematode community changes induced by continuous cropping in strawberry habitat. The decreased *H*′ of nematode fauna in 5-Y and 7-Y reflects increases in specific plant-feeding nematodes associated with strawberry plants, which results from continuous physical disturbance. On the other hand, the slightly increased *Ig* and *TD* in 7-Y reflected the decreases of some omnivores and rare genera, such as *Dorylaimus, Protorhabditis*, and *Wilsonema*, together with the increase in some fungivores and herbivore nematodes, i.e., *Aphelenchoides, Tylenchus*, and *Tetylenchus*. These changes could be attributed to the continuous addition of nitrogen via fertilizer decreased total nematode abundance and diversity, but responses varied among trophic groups^76^. Besides, the sharp decrease in omnivores/predators, probably because these two groups, as K-strategists, are sensitive to environmental change and correlated negatively with rich nutrient resources. This result is in line with other studies, which indicated that omnivores/predators are apt to be negatively affected by nutrient additions^77^, and tend to be less abundant in arable fields^78^, especially in long-term farming systems^71^, compared with natural areas. However, declining diversity indices indicated the degraded ecosystem stability and quality with increasing continuous cropping years.

Nematodes that feed on bacteria and fungi are associated with decomposition and nutrient mineralization processes^79^,^80^. Higher densities of bacterivores and fungivores, together with higher *BI* value may indicate increased rates of decomposition, possibly resulting in improved mineralization of nitrogen and other nutrients in 0-Y and 2-Y, and vice versa from 3-Y to 7-Y. The *NCR*, as well as *CI*, can describe contributions of the above trophic groups to decomposition processes. In 0-Y and 2-Y, NCR, with variation between 1 (bacterial feeding nematode dominance) and 0 (fungal feeding nematode dominance), indicated that bacterivores were dominant. This in conjunction with lower *CI* indicates the predominant contribution of bacterial feeders to decomposition and relatively quick turnover of the available organic matter^33^,^81^. As years in continuous cropping increased from 3-Y to 7-Y, changes in soil conditions resulted in a switch to the fungal pathway, possibly together with a slower rate of organic matter turnover. Moreover, higher proportions of fungal-feeding nematodes probably reflected more favorable soil microhabitats for fungi^69^, which is consistent with previous studies that showed continuous cropping of strawberry could induce significant increases in fungal populations^82^, especially some pathogenic fungi which could cause serious disease and play a key role in strawberry continuous-cropping problems^83^.

In general, the ecological function indices showed no discernible pattern as the years of continuous cropping increased, which reflects the similar level of disturbance to the soil environment among the different continuous-cropping years. According to the faunal analysis profile, all continuous-cropping strawberry sites located into Quadrant A, which indicates a high level of disturbance, highly N-enriched, bacterial decomposition channels and a low C: N ratio of the soil web. There were also higher *EI* values (*EI >* 50), common in agricultural food webs^33^,^84^,^85^, indicated the available resources were enriched, specifically manifesting in higher soil fertility levels, better nutrient availability, etc. *SI* represents time course progressions in the structure of the soil food web, primarily evaluated by omnivores and predator nematodes which are sensitive to disturbance and stress^23^. Also, there were no significant differences observed on the *SI* values among the differently aged ecosystems, due to their similar practical management, with low *SI* values in the repeated tillage and other disturbed arable agroecosystems^33^,^51^,^86^. In addition, the repeated action of replanting strawberry seedlings caused some disturbance of the food web, which continuously worsened as years progressed.

## Conclusions

Soil nematodes dominance was distinctly different among different years of continuous-cropping strawberry, but the genus composition was similar due to because of due to the local pedogenesis and species pool, as well as the same plant habitat and same agronomic management. As the years of continuous-cropping increased, soil pH significantly decreased both because of accumulated phenolic acids and nitrogen inputs that exceeded what could be processed by soil microorganisms. In addition to the phytotoxicity of accumulated phenolic acids in replant ecosystem, we speculated that inhibition of root ion uptake due to low soil pH was partly responsible for the plant weakness. Moreover, nutrient resources and phenolic acids were the main parameters in structuring the patterns of soil nematode community. Along with increasing continuous-cropping years, increased concentrations of phenolic acids in soil are toxic against both *Acrobeloides* and *Rhabditis*, thus bacterivorous nematode population didn’t show explosive response to the increased organic matter in this study. On the contrary, the increased fungivore populations, especially the less specialized-feeding *Aphelenchoides,* indicated possible increases both in strawberry nematodes and pathogenic fungal populations which harm to strawberry plants. In general, the unbalanced condition of soil nutrients negatively impacted the nematode community and ecosystem stability. Specifically, the changed dominant decomposition method (bacterial to fungal) as well as the disturbed soil food web and the degraded available nutrient conditions could be reflected by nematode community.

Overall, this study provides an early step towards understanding how changes in soil conditions and nematode community structure can contribute to explaining the bases of replant problem from the view of soil fauna, and emphasizes nematode assembly a strong role of predictability and niche structuring in soil with repeated action of planting strawberry seedlings.

## Additional Information

**How to cite this article**: Li, X. *et al*. Effects of long-term continuous cropping on soil nematode community and soil condition associated with replant problem in strawberry habitat. *Sci. Rep.* **6**, 30466; doi: 10.1038/srep30466 (2016).

## Figures and Tables

**Table 1:** The absolute abundance (per 100 g dry soil) of various nematodes identified from different continuous-cropping years of strawberry greenhouses averaged over the strawberry growing season.

Family	Genera	Guild*	Dominance				
			0-YCC	2-YCC	3-YCC	5-YCC	7-YCC
Diplogasteridae	*Diplogaster*	Ba_1_	33.08 i**	25.65 i	24.21 i	10.37 i	0.00−
Panagrolaimidae	*Panagrolaimus*	Ba_1_	27.62 i	23.91 i	10.45 i	0.00−	0.00−
Rhabditidae	*Diploscapter*	Ba_1_	1006.18 v	830.50 v	891.44 v	697.97 v	395.91 iv
	*Neorhabditis*	Ba_1_	14.47 i	10.69 i	0.00−	0.00−	0.00−
	*Protorhabditis*	Ba_1_	27.62 i	22.31 i	12.82 i	0.00−	0.00−
	*Rhabditis*	Ba_1_	936.67 v	890.76 v	883.96 v	902.57 v	837.48 v
Cephalobidae	*Acrobeles*	Ba_2_	35.60 i	22.18 i	0.00−	0.00−	0.00−
	*Acrobeloides*	Ba_2_	996.98 v	906.53 v	347.86 iv	162.93 iii	59.08 ii
	*Cephalobus*	Ba_2_	34.56 i	24.18 i	18.04 i	17.99 i	25.82 i
	*Cervidellus*	Ba_2_	11.96 i	0.00−	0.00−	0.00−	0.00−
	*Eucephalobus*	Ba_2_	605.57 v	645.67 v	0.339.84 iv	312.01 iv	291.54 iv
Monhysteridae	*Monhystera*	Ba_2_	19.35 i	20.57 i	9.50 i	0.00−	0.00−
0.00Plectidae	*Plectus*	Ba_2_	17.28 i	25.52 i	16.86 i	9.20 i	0.00−
	*Wilsonema*	Ba_2_	18.61 i	15.50 i	0.00−	0.00−	0.00−
Prismatolaimidae	*Prismatolainus*	Ba_3_	51.55 ii	43.82 ii	0.00−	0.00−	0.00−
Alaimdae	*Alaimus*	Ba_4_	59.08 ii	5.74 i	0.00−	0.00−	0.00−
	**Bacterivores**		**3897** ± 121 a***	**3565** ± **67 b**	**2554** ± **58 c**	**2113** ± **46 d**	**1609** ± **24 e**
Aphelenchoididae	*Aphelenchoides*	Fu_2_	77.98 ii	61.06 ii	69.56 ii	114.90 iii	334.91 v
Aphelenchidae	*Aphelenchus*	Fu_2_	34.86 i	28.05 i	58.28 i	65.60 iii	121.09 iii
	**Fungivores**		**114** ± **10 c**	**89** ± **8 d**	**128** ± **18 c**	**179** ± **28 b**	**456** ± **5 a**
Dorylaimidae	*Dorylaimus*	Po_4_	71.78 ii	212.23	56.5	34.39	17.18
Mononchidae	*Mononchus*	Po_4_	26.73 i	18.17 i	18.52 i	0.00−	0.00−
	**Omniovores/Predatores**		**98** ± **11 b**	**231** ± **4 a**	**75** ± **4 c**	**34** ± **13 d**	**17** ± **5 e**
Paratylenchidae	*Paratylenchus*	PF_2_	27.62 i	28.99 i	248.60 iv	207.05 iv	114.89 iii
Tylenchidae	*Tetylenchus*	PF_2_	145.63 iii	53.44 ii	316.72 iv	261.41 iv	218.56 iv
	*Tylenchus*	PF_2_	98.07 iii	66.44 iii	67.30 ii	254.24 iv	429.57 v
Heteroderidae	*Meloidogyne*	PF_3_	0.00−	0.00−	31.93 i	35.97 ii	65.34 iii
Hoplolaimidae	*Helicotylenchus*	PF_3_	0.00−	0.00−	0.00−	0.00−	1.19 i
	*Rotylenchus*	PF_3_	4.28 i	1.87 i	0.00−	2.96 i	0.79 i
Pratylenchidae	*Pratylenchus*	PF_3_	28.06 i	19.37 i	68.85 ii	34.49 ii	57.49 ii
Tylenchorhynchidae	*Tylenchorhynchus*	PF3	19.79 i	15.79 i	67.54 ii	50.04 ii	119.26 iii
**Plant-feeders**			324 ± 11 c	186 ± 23 d	801 ± 40 b	846 ± 29 b	1007 ± 61 a
**Total**			**4833** ± **219 a**	**4008** ± **136 b**	**3558** ± **86 c**	**3173** ± **78 cb**	**3089** ± **75 d**

^*^Ba, bacterivore; Fu, fungivore; Po, predators/omniovores; PF, plant feeders. Suffix numbers are c-p value for the taxa.

^**^v: Eudominance (≥10%); iv: Dominance (5–10%); iii: Subdominance (2–5%); ii: Resident (1–2%); i: Subresident (0–1%); -Absent (0%).

^***^Significant values (Mean ± SD) in the same row followed by identical alphabets do not differ significantly (P < 0.05) in ANOVA and Duncan tests.

**Table 2:** Nematicidal activity of phenolic acids against *Rhabditis* spp. and *Acrobeloides* spp.

Phenolic acids	Nematodes	Regression equation	Correlation coefficient	LC_50_(μg/mL)
Cinnamic acid	*Rhabditis*	y = 1.495 × − 3.506	0.966	221.412
	*Acrobeloides*	y = 2.466 × −5.043	0.985	110.903
Ferulic acid	*Rhabditis*	y = 1.327 × −3.301	0.961	307.208
	*Acrobeloides*	y = 2.107 × −4.612	0.977	154.412
p-Coumaric acid	*Rhabditis*	y = 1.304 × −3.427	0.951	425.156
	*Acrobeloides*	y = 1.602 × −3.978	0.978	303.485
p-Hydroxybenzoic acid	*Rhabditis*	y = 1.260 × −3.208	0.958	351.852
	*Acrobeloides*	y = 1.949 × −4.540	0.989	213.663
